# Hepatitis B virus reactivation sustained by a hepatitis B virus surface antigen immune-escape mutant isolate in a patient who was hepatitis B core antibody positive during treatment with sofosbuvir and velpatasvir for hepatitis C virus infection: a case report

**DOI:** 10.1186/s13256-019-2232-3

**Published:** 2019-09-22

**Authors:** Luca Foroghi Biland, Ludovica Ferrari, Vincenzo Malagnino, Elisabetta Teti, Carlotta Cerva, Adele Gentile, Marianna Aragri, Romina Salpini, Valentina Svicher, Massimo Andreoni, Loredana Sarmati

**Affiliations:** 1 Infectious Diseases Clinic, Policlinico Tor Vergata, Rome, Italy; 2 Department of Experimental Medicine and Surgery, University of Rome Tor Vergata, Rome, Italy; 3 Department of System Medicine, Tor Vergata University of Rome, Rome, Italy

**Keywords:** Hepatitis B virus, HBV reactivation, Direct acting antivirals, Coinfection HBV/HCV, Immune-escape HBV mutant

## Abstract

**Background:**

Although several cases of hepatitis B virus reactivation have been described in patients with a history of hepatitis B virus infection while undergoing treatment for hepatitis C virus infection with direct acting antivirals, the question of whether hepatitis B virus surface antigen immune-escape mutations might play a role has not been addressed so far.

**Case presentation:**

We report a case of hepatitis B virus reactivation in a Caucasian patient infected with hepatitis C virus during treatment with sofosbuvir and velpatasvir. A 50-year-old man with a genotype 1a hepatitis C virus infection was considered for therapy. His serological profile was hepatitis B virus surface antigen-negative, hepatitis B virus core antibody-positive, hepatitis B virus surface antibody-negative, and anti-hepatitis D virus-positive. The detection of hepatitis B virus deoxyribonucleic acid (DNA) indicated active viral replication during the direct acting antiviral treatment that spontaneously returned to undetectable levels after treatment completion. Starting from week 12 after the end of treatment, hepatitis B virus surface antibody titers and hepatitis B virus e antibody developed. Sequencing analysis revealed the hepatitis B virus genotype D3 and the presence of two relevant immune-escape mutations (P120S and T126I) in the major hydrophilic region by analyzing the S region.

**Conclusions:**

We speculate that the presence of the hepatitis B virus surface antigen mutations, endowed with the enhanced capability to elude the immune response, could play a role in hepatitis B virus reactivation. This observation confirms that occult hepatitis B infection should also be carefully monitored, through surveillance of the hepatitis B virus viral load before and during direct acting antiviral treatment of hepatitis C virus.

## Background

Chronic infection with either hepatitis B virus (HBV) or hepatitis C virus (HCV) is responsible for a high morbidity and mortality burden worldwide. Since these viruses share common transmission routes, HBV and HCV coinfection is not rare, and it is estimated that 1–15% of patients with HCV infection might be coinfected with HBV [1]. In developed countries, people who use drugs (PWUD) are particularly exposed to HBV-HCV coinfection, and epidemiological studies suggest that approximately half of the PWUD who are HCV-positive may have an occult HBV infection (OBI) [2]. OBI, defined by the detection of HBV deoxyribonucleic acid (DNA) in hepatocytes or in the serum of patients who are hepatitis B virus surface antigen (HBsAg)-negative, can contribute to accelerated evolution of cirrhosis or the development of hepatocellular carcinoma (HCC) in patients coinfected with HCV [3, 4]. The interaction between HBV and HCV can give rise to a wide spectrum of virological patterns [5]; in the majority of cases, HCV predominates over HBV replication and transcription via immunological and virological mechanisms [5, 6]. Recently, several cases of HBV reactivation (HBVr) [7, 8] have been described in patients with a history of HBV infection while undergoing treatment for HCV infection with direct acting antivirals (DAAs), suggesting that the clearance of HCV abrogates the inhibitory effect exerted by HCV on HBV replication. Despite increasing awareness of the risk of DAA-induced HBVr, the question of whether HBsAg immune-escape mutations might play a role in this process has not been addressed so far. Previous studies have shown that immune-escape mutations can play a role in HBV reactivation driven by immunosuppression [9]. Here, we report a case of HBVr in a patient infected with HCV, with resolved HBV infection, treated with sofosbuvir and velpatasvir for 12 weeks. Surprisingly, the patient had reactivated HBV infection and the virus developed two mutations localized in an immune-active HBsAg region, thus making ineffective HBV-specific immune response and favoring HBV viral load (VL) flare. The clinical relevance of the case is also due to the difficulty in the diagnosis of HBVr since the mutations in HBsAg did not allow its detection by the usual laboratory tests.

## Case description

A Caucasian 50-year-old man, in a stable relationship and employed, was considered for HCV treatment at our center. In 1996, during a hospitalization for jaundice, he received a diagnosis of acute hepatitis B/hepatitis D virus (HDV) infection. At that time, an HCV infection genotype 1a was also diagnosed, which had never been treated. The patient had a hystory of previous use of injected heroin and inhaled cocain, and he was in opiate substitution therapy (OST) with buprenorphine. Except for this, he did not use other drugs. He smoked tobacco, reported a previous alcohol abuse, and at the first assessment he stated that he drank 1–2 drinks a day. His medical history was notable for a sinus tachycardia and he underwent inguinal hernioplasty and appendectomy. No liver disease was documented in his family history.

His serological profile at admission to our center was HBsAg-negative, HBV core antibody (anti-HBc)-positive, HBV surface antibody (anti-HBs)-negative, and hepatitis D virus antibody (anti-HDV)-positive.

On presentation, he did not report any symptoms and denied previous episodes of ascites, hematemesis, melena, hepatic encephalopathy, and vomiting. A physical examination excluded signs of hepatic decompensation; in particular, it did not reveal ascites, splenomegaly, leg swelling, jaundice, and spider angiomas. The remainder of the examination was normal.

In the HCV pre-treatment assessment, he had F0–F1 fibrosis stage (Metavir score) evaluated by transient elastography (median liver stiffness 6.9 kPa) and an abdominal ultrasound excluded the presence of HCC and signs of portal hypertension. Laboratory analysis showed that alanine aminotransferase (ALT) was 51 IU/L, aspartate aminotransferase (AST) was 52 IU/L, and glucose 108 mg/dL. The count of white cells and platelets, and levels of hemoglobin, creatinine, alpha-fetoprotein, and electrolytes were normal; liver function tests were normal. HCV VL was 7,014,213 IU/ml and no HCV resistance-associated substitutions were found. His FIB-4 score was 2.13. Other laboratory test results are shown in Table 1; data regarding the HBV VL were not available.

**Table 1:** Laboratory data at baseline

Laboratory data	
Hemoglobin (g/dl)	13.6
Hematocrit (%)	41.2
White blood cells (10^3^/mm^3^)	6.74
Neutrophils (10^3^/mm^3^)	4.5
Lymphocytes (10^3^/mm^3^)	1.6
Platelet count (10^3^/mm^3^)	170
Aspartate aminotransferase (IU/l)	52
Alanine aminotransferase (ALT) (IU/l)	51
Gamma-glutamyltransferase (GGT) (IU/l)	31
Alpha-fetoprotein (IU/ml)	< 1
Total bilirubin (mg/dL)	0.34
Direct bilirubin (mg/dl)	0.13
Alkaline phosphatase (IU/L)	63
Sodium (mEq/L)	139
Potassium (mEq/L)	4.3
Creatinine (mg/dl)	1
eGFR (CKD-EPI)	79.5
Glucose (mg/dl)	108
Glycated hemoglobin (mmol/mol)	35
Albumin (g/dl)	4.26
HCV-RNA (IU/ml)	7,014,213
anti-HDV	positive
anti-HIV	negative
Venereal Disease Research Laboratory (VDRL)	negative
Tuberculosis (TB)-interferon gamma release assay (IGRA)	negative
Prothrombin time – International normalized ratio (INR)	0.94
HBsAg	negative
anti-HBs	negative
total anti-HBc	positive
anti-HBc IgM	negative
HBeAg	negative
anti-HBe	negative

Hepatitis B virus e antigen, hepatitis B virus e antibody, and hepatitis B virus core antibody IgM were performed at week 8 of treatment; hepatitis D virus antibody was performed at week 8 after the end of treatment. *anti-HBc* hepatitis B virus core antibody, *anti-HBe* hepatitis B virus e antibody, *anti-HBs* hepatitis B virus surface antibody, CKD-EPI Chronic Kidney Disease Epidemiology Collaboration, *eGFR* estimated glomerular filtration rate, *HBeAg* hepatitis B virus e antigen, *HBsAg* hepatitis B virus surface antigen, *HCV* hepatitis C virus, *HDV* hepatitis D virus

**Fig. 1:**
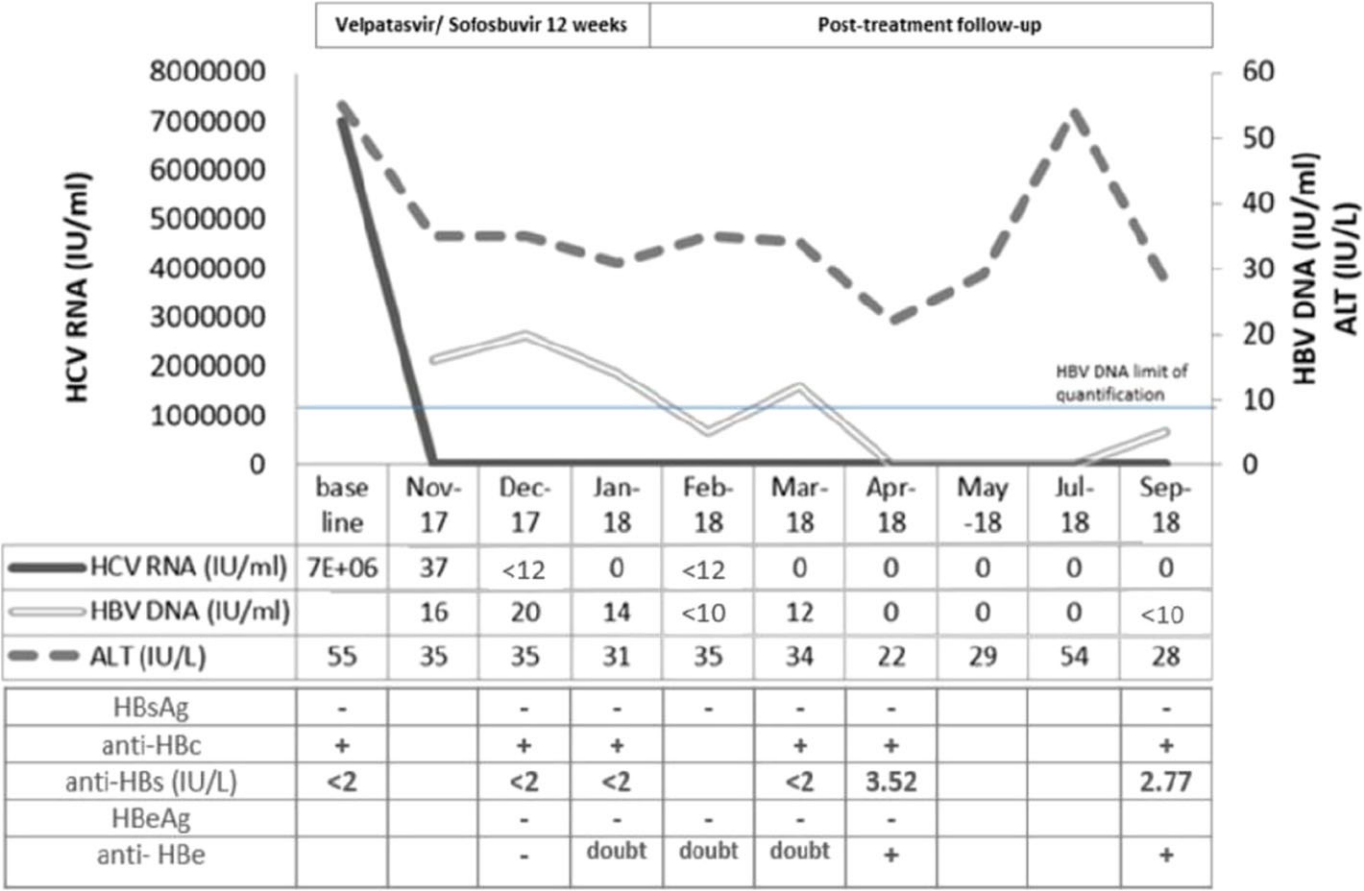
Virological and biochemical parameters trend during and after direct acting antiviral treatment. Hepatitis B virus surface antibody, hepatitis B virus surface antigen, hepatitis B virus e antibody, hepatitis B virus e antigen, and hepatitis B virus core antibody results are presented corresponding to the time-point of collection. *ALT* alanine aminotransferase, *anti-HBc* hepatitis B virus core antibody, *anti-HBe* hepatitis B virus e antibody, *anti-HBs* hepatitis B virus surface antibody, *HBeAg* hepatitis B virus e antigen, *HBsAg* hepatitis B virus surface antigen, *HBV* hepatitis B virus, *HCV* hepatitis C virus

In October 2017, he started once daily sofosbuvir/velpatasvir (400/100 mg) without ribavirin (RBV) for 12 weeks. HCV ribonucleic acid (RNA) was 37 IU/ml at week 4, below the limit of quantification (< 12 IU/ml) at week 8 and undetectable at the end of treatment (EOT) (Fig. 1). The detection of HBV DNA showed active viral replication and values of 16, 20, and 14 IU/ml were found at weeks 4, 8, and 12, respectively, of DAA treatment. Of note, the HBsAg and hepatitis B virus e antigen (HBeAg) remained negative despite evidence of ongoing HBV replication. The sequencing analysis of the HBV reverse transcriptase (RT)/S regions revealed the presence of the HBV genotype D3 and the absence of amino acid substitutions conferring resistance to any anti-HBV drugs. In contrast, by analyzing the S region, two relevant mutations, P120S and T126I, localized in the major hydrophilic region, an immune-active HBsAg domain, were found. He reached a sustained virological response (SVR) for HCV at 12 and 24 weeks after the EOT. An abdomen ultrasound performed at week 24 after the EOT showed hepatic steatosis but excluded the presence of nodular lesions. At the same time-point, non-invasive measurement of liver fibrosis, through transient elastography, confirmed the starting fibrosis stage (median liver stiffness 5.0 kPa, F0/F1 Metavir stage). At weeks 4 and 12 after the EOT, the HBV VL, in the absence of antiviral therapy, was < 10 IU/ml and 12 IU/ml, respectively, but returned below the limit of quantification at weeks 16, 20, and 24 after the EOT. A search for HDV RNA, performed at week 8 after the EOT, was negative. HBsAg and HBeAg remained negative despite the HBVr at all tested time-points; however, starting from week 12 after the EOT, the anti-HBs and hepatitis B virus e antibody (anti-HBe) titers spontaneously developed.

## Discussion and conclusions

To the best of our knowledge, we are the first to report a case of HBVr characterized by the emergence of viral strains with immune-escape mutations in a patient coinfected with HCV with isolated anti-HBc positivity and on a successful treatment with the latest generation of DAAs sofosbuvir and velpatasvir. HBVr is characterized by a sudden increase in HBV replication (> 2 log_10_ in viremic patients or detectable HBV DNA in patients with resolved HBV infection) or a reversion to an HBsAg-positive status in individuals who are HBsAg-negative and has been extensively reported in patients treated with immunosuppressive agents and/or intensive chemotherapy [10]. HBVr during chronic HCV infection (CHC) was also previously described in patients treated with interferon-based therapies [11], but recent reports demonstrated that HBVr is more frequent and occurs earlier in patients with CHC treated with DAAs. Moreover, a meta-analysis [7] showed that the overall risk of HBVr was 24% in patients who were HBsAg-positive and 1.4% in those with resolved HBV infection. Although HBVr is not uncommon, the occurrence of accompanying hepatitis in patients with resolved HBV infection is a rare event [12–14] and only occasionally leads to clinically significant outcomes. According to these previous reports, we observed an early HBV viremia in the absence of a concomitant increase in the hepatic necro-inflammatory markers, which in the following months returned spontaneously below the limit of quantification. Some elements lead us to hypothesize reactivation. First, the viral decay kinetics, and second, the antibody response (anti-HBs and anti-HBe), which developed starting from week 12 after the EOT, were probably induced by the presence of a singular episode of HBV flare. In addition, our patient was also coinfected with HDV, which is known to exert a suppressive role on the replication of HBV [15] and may have prevented a further rise in the HBV viremia. According to the American Association for the Study of Liver Diseases-Infectious Diseases Society of America (AASLD-IDSA) recommendations [16], patients who are HBsAg-positive meeting criteria for treatment of active HBV infection should be started on therapy at the same time or before HCV DAA therapy is initiated, whereas patients with low or undetectable HBV DNA titers can either receive prophylactic treatment or be monitored at regular intervals. Conversely, the same guidelines do not provide clear recommendations for the monitoring of HBV DNA among patients with resolved HBV infection. Despite the lack of indications from the current guidelines, we decided to search for HBV DNA at week 4, in the absence of an increase of transaminases, because of the European Medicines Agency (EMA) warning on the risk of early reactivation in patients with overt HBV infection or OBI (warning letter EMA/795452/2016, 2 December 2016). Furthermore, as reported above, the increase of transaminases related to HBV reappearance is not so frequent; hence, only monitoring the level of transaminases would not be sufficient.

An element of great interest is that, to the best of our knowledge, this is the first report of HBVr characterized by the emergence of viral strains carrying immune-escape mutations in a patient coinfected with HCV with isolated anti-HBc positivity and on successful treatment with the latest generation of DAAs. Immune-escape mutations in HBsAg are clinically relevant since their presence, by enabling the evasion of the adaptive immune response, can promote both viral replication [17] and chronic persistence of HBV infection [18, 19] and may reduce HBsAg diagnostic detection. Interestingly, the presence of the immune-escape mutants was related to HBVr in patients who had undergone immunosuppressive treatments [9]. In recent years, only two case reports have focused on HBVr of the HBsAg mutant strains in patients treated with DAAs for HCV [13, 18]. In the first case [13], genome sequencing revealed a T118K mutation in the S region, but in contrast to our description, the HBV viremia occurred 5 months after the EOT with daclatasvir and asunaprevir. Notably, the serological profile of this patient at baseline was not reported. More recently, Fusco *et al.* [20] described a reactivation of vaccine-escape HBV mutants during anti-HCV treatment with ledipasvir/sofosbuvir (90/400 mg) for 12 weeks. Similar to our case, the patient had a baseline profile of OBI infection (HBsAg-negative, anti-HBc-positive, HBV DNA 120 UI/L), but, in contrast to our patient, the anti-HBs levels were above the protective threshold before starting the treatment. A limitation of our case description is the lack of measurement of the HBV DNA levels before the start of the DAA treatment, in line with the latest European Association for the Study of the Liver (EASL) recommended guidelines; hence, we cannot exclude pre-existing low-level HBV viremia in the context of OBI.

To conclude, we speculate that the presence of the antigen S mutations, endowed with enhanced capability to elude immune response, could play a role in HBVr. This observation confirms that OBI should also be carefully monitored, through the surveillance of HBV VL, before and during DAA treatment of hepatitis C. This approach could enable early identification of HBVr and find a pre-existing detectable HBV viremia in the context of OBI, which may be at higher risk of reactivation. Further studies are warranted to confirm the hypothesis regarding the role of HBsAg immune-escape mutants in predisposing HBVr during DAA regimens for CHC.

## Data Availability

The datasets used and analyzed during the current study are available from the corresponding author on reasonable request.

## Abbreviations

AASLD-IDSA: American Association for the Study of Liver Diseases-Infectious Diseases Society of America
ALT: Alanine aminotransferase
anti-HBc: Hepatitis B virus core antibody
anti-HBe: Hepatitis B virus e antibody
anti-HBs: Hepatitis B virus surface antibody
anti-HDV: Hepatitis D virus antibody
AST: Aspartate aminotransferase
CHC: Chronic hepatitis C virus infection
DAAs: Direct acting antivirals
EASL: European Association for the Study of the Liver
EMA: European Medicines Agency
EOT: End of treatment
HBeAg: Hepatitis B virus e antigen
HBsAg: Hepatitis B virus surface antigen
HBV: Hepatitis B virus
HBVr: Hepatitis B virus reactivation
HCC: Hepatocellular carcinoma
HCV: Hepatitis C virus
HDV: Hepatitis D virus
OBI: Occult hepatitis B infection
OST: Opiate substitution therapy
PWUD: People who use drugs
RBV: Ribavirin
RT: Reverse transcriptase
SVR: Sustained virological response
VL: Viral load
